# Characterization of Endolysin LysG77YL from *Bacillus licheniformis*-Infecting Bacteriophage G77YL and Application as an Antimicrobial Agent

**DOI:** 10.4014/jmb.2601.01064

**Published:** 2026-03-25

**Authors:** Nanjoo Park, Yerin Cho, Yerin Kang, Minsuk Kong

**Affiliations:** 1 Division of Food Technology & Nutrition, SunMoon University, Asan 31460, Republic of Korea; 2 Department of Food Science and Biotechnology, Seoul National University of Science and Technology, Seoul 01811, Republic of Korea

**Keywords:** *Bacillus licheniformis*, Bacteriophage, Endolysin, Dairy product contamination

## Abstract

*Bacillus licheniformis* is a spore-forming, Gram-positive bacterium widely found in nature and frequently associated with spoilage in dairy products due to its extracellular enzyme production. To develop a targeted biocontrol strategy, a novel lytic phage, G77YL, was isolated from the Gyeongchun Line Forest Trail in South Korea. Host range analysis revealed that G77YL specifically infects several strains of *B. licheniformis*. Genome analysis indicated that it is a virulent phage devoid of lysogeny-related genes and encodes a glycosyl hydrolase family 25 endolysin, designated as LysG77YL. The endolysin (LysG77YL) and its enzymatically active domain (LysG77YL_EAD) were cloned, expressed, and purified. Lytic assays demonstrated that LysG77YL_EAD exhibited broader and more potent antibacterial activity than the full-length enzyme, targeting all *Bacillus* strains tested, including eight *B. licheniformis* strains, while showing no activity against non-target Gram-positive and Gram-negative bacteria. Stability testing showed that LysG77YL_EAD retained high lytic activity under various pH (7-9), salt (100-200 mM NaCl), and temperature (4-40°C) conditions. In a food application using sterilized milk contaminated with *B. licheniformis*, treatment with 4 μM LysG77YL_EAD resulted in a 4 log CFU/ml reduction in bacterial count within 30 min at 4°C. These findings highlight the potential of LysG77YL_EAD as a stable and efficient endolysin-based biocontrol agent for controlling *B. licheniformis* contamination in dairy products, providing an attractive alternative to conventional antimicrobial agents.

## Introduction

*Bacillus licheniformis* is a spore-forming, Gram-positive bacterium that is widely distributed in natural environments such as soil and water. *B. licheniformis* is extensively utilized in industrial applications for the production of enzymes, such as proteases, amylase, and antimicrobial compounds [1]. Despite its beneficial uses, this organism has also been implicated in the spoilage of dairy products due to its secretion of extracellular enzymes, including proteases, lipases, and β-galactosidases. This enzymatic activity contributes to the deterioration of product quality and a reduction in shelf life [2]. Although the role of *B. licheniformis* toxins in foodborne illness has not been clearly established, toxin-producing strains have been isolated from food products associated with food poisoning cases, including raw milk and milk powder [3, 4]. Given its capacity to produce toxins, *B. licheniformis* has the potential to cause foodborne illnesses. To control *B. licheniformis* contamination, traditional methods, such as heating, high-pressure, irrigation, and chemical agents are commonly used in the industry [5, 6]. However, these methods often reduce food quality and may pose toxicological risks [7].

Bacteriophages (phages) are viruses that infect bacteria and specifically kill their host using lysis. They show host specificity, remaining harmless to humans and other organisms [8]. Notably, several phages have been recognized as safe by the US Food and Drug Administration (FDA), having obtained Generally Recognized as Safe (GRAS) status, and are applied in the food industry as natural antibacterial agents [9]. Despite their potential, phages typically exhibit a narrow host range, meaning that a cocktail of multiple phages is necessary for effective application. Additionally, the use of term such as ‘viruses’ or ‘virulent phage’ in food products may lead to negative consumer perceptions [10]. To address these limitations, phage-derived peptidoglycan hydrolases, known as endolysins, have recently been recognized as promising alternative antibacterial agents [10].

Endolysins are peptidoglycan-degrading enzymes that are released by bacteriophages during the terminal phase of the lytic cycle to facilitate host cell lysis [11]. In general, endolysin derived from bacteriophage that infect Gram-positive bacteria typically possesses a two-domain structure, comprising an N-terminal enzymatically active domain (EAD) responsible for host cell wall lysis and a C-terminal cell wall binding domain (CBD) that enables specific attachment to the host cell wall [12]. These features have enabled the use of EADs and CBDs in the biocontrol and detection agents of target bacteria [13]. Endolysins offer several advantages as antibacterial agents, as they act rapidly and resistance has not been reported to date [14]. As they specifically target the peptidoglycan of prokaryotes, they are harmless to humans, animals, and plants. Additionally, their genus-level specificity minimizes disruption to the normal microbiota [10]. Consequently, several endolysins were recently granted GRAS status (GRN 802) by the US FDA [15]. Despite the significant potential of endolysins as biocontrol agents, to date, only a few studies have been characterized, and research regarding the application of endolysins to control *B. licheniformis* remains highly limited.

In this study, a novel virulent *B. licheniformis* bacteriophage, G77YL, was isolated, and its biological and genomic characteristics were analyzed. An endolysin, LysG77YL, was identified within the G77YL genome and further characterized. LysG77YL and its enzymatically active domain (LysG77YL_EAD) exhibited a broader antibacterial spectrum than the parental phage. Notably, LysG77YL_EAD showed stronger lytic activity against *B. licheniformis* compared to LysG77YL. Furthermore, the antimicrobial efficacy of LysG77YL_EAD against *B. licheniformis* was evaluated in a food matrix. To the best of our knowledge, although bacteriophages have been investigated for controlling *B. licheniformis* in dairy products [16], the application of endolysin has not yet been reported. These findings highlight the potential of LysG77YL_EAD as an effective biocontrol agent for managing *B. licheniformis* contamination in the food industry.

## Materials and Methods

### Bacterial Strains and Growth Conditions

The bacterial strains used in this study are summarized in Table 1. *Bacillus licheniformis* KACC 18777 was used as the host and propagation strain for the bacteriophage G77YL. The strain was obtained from the Korean Agricultural Culture Collection (KACC), Rural Development Administration (RDA), Jeonju, Republic of Korea. All *B. licheniformis* strains, along with other Gram-positive bacteria such as *Bacillus* and *Listeria* species, were cultured in Brain Heart Infusion (BHI) broth (BD Difco, USA) overnight at 37°C. *Clostridium perfringens* strains were cultivated anaerobically in BHI broth under the same temperature conditions. Unless otherwise noted, bacteria cells were grown in Luria-Bertani (LB) broth (BD Difco) overnight at 37°C.

### Bacterial Isolation and Propagation

*B. licheniformis*-infection bacteriophage G77YL was isolated from the Gyeongchun Line Forest Trail in Seoul, South Korea. The isolation procedure was based on a previously established method [17], with minor modifications. To amplify phage G77YL, serial propagation was performed, followed by precipitation using 10% polyethylene glycol 6000 (Junsei, Japan). The phage particles were then harvested by centrifugation (15,000 ×*g*, 20 min, 4°C 1736R, LABOGENE, Republic of Korea), resuspended in SM buffer, and further purified using CsCl gradient ultracentrifugation (78,500 ×*g*, 2 h, 4°C; CP80NX, Himac, Japan). The resulting concentrated phage preparation was dialyzed against 1L of SM buffer using 3.5 kDa MWCO SnakeSkin Dialysis Tubing (Thermo Fisher Scientific, USA).

### Morphological Analysis

The morphology of G77YL was analyzed using transmission electron microscopy (LIBRA 120, Carl Zeiss, Germany). Purified bacteriophage samples (1 × 10^10^ PFU/ml in SM buffer) were dropped onto a carbon-coated copper grid (Formvar/Carbon 200 mesh, EMS) and incubated at room temperature for 1 min. The remaining sample was gently removed by blotting, and the grid was subsequently subjected to negative staining with 2% uranyl acetate (pH 4.0, EMS) for 20 sec. Following air drying, imaging of the G77YL phage was performed using an Energy-Filtering Transmission Electron Microscope (EF-TEM; LIBRA 120, Carl Zeiss, Germany) operated at an accelerating voltage of 120 kV [18]. Bacteriophage G77YL was classified into their relative family according to the International Committee on Taxonomy of Viruses (ICTV) classification [19].

### Host Range Test

The bacterial strains used for host range test are listed in Table 1. G77YL phage suspensions were prepared by 10-fold serial dilutions, and 10 μl of each dilution was spotted onto 0.7% LB soft agar overlays containing different bacterial strains. The plates were incubated overnight at 37°C, and phage titers were determined based on the number of plaque-forming units (PFU/ml).

### Whole-Genome Sequencing and Genomic Analysis

Bacteriophage genomic DNA was extracted using phenol-chloroform extraction, and concentrated by ethanol precipitation as described previously [20]. Purified phage genomic DNA was sequenced using the Illumina MiSeq platform (Sanigen Inc., Republic of Korea), and the genome was assembled *de novo* using the CLC Genomics Workbench version 10.0.1. The identification of open reading frames (ORFs) was initially performed using the Rapid Annotation using Subsystem Technology (RAST) pipeline [21]. Conserved protein domains were identified via InterProScan [22] and the Conserved Domain Database (CDD) provided by NCBI. To predict the functions of open reading frames (ORFs), Basic Local Alignment Search Tool for proteins (BLASTP) searches were conducted [23]. The identification of tRNA genes was performed using the tRNAscan-SE tool [24]. The genome was visualized with the CGView server [25], and the complete sequence of *B. licheniformis* bacteriophage G77YL has been deposited in GenBank (Accession number PX311066). A phylogenetic analysis was performed to explore the evolutionary relationships among *Bacillus* phages. For this analysis, 13 *Bacillus* phage genomes were obtained from NCBI database, and their amino acid sequences were aligned using ClustalW. The phylogenetic tree was constructed using the maximum likelihood method and visualized with MEGA11 software [26]. The protein structure of endolysin LysG77YL was predicted by AlphaFold2 [27].

### Cloning, Expression, and Purification

The plasmids and primers used in this study are listed in Table S1. The endolysin gene (LysG77YL) and its EAD (LysG77YL_EAD) were amplified from the genomic DNA of the phage G77YL by polymerase chain reaction (PCR). The gene encoding LysG77YL and LysG77YL_EAD, digested with BamHI and HindIII restriction enzymes was inserted into the pET28a vector (Novagen, USA), which has maltose binding protein (MBP) and N-terminal hexahistidine (His)-tag sequence. The recombinant plasmid was transformed into competent *E. coli* BL21 (DE3) for protein expression. Protein expression was induced by adding 0.5 mM isopropyl β-D-1-thiogalactopyranoside (IPTG; Sigma-Aldrich, USA) when the bacterial culture reached an OD_600_ of 0.6-0.8, followed by incubation at 18°C with shaking for 20 h. Cells were harvested by centrifugation at 4,000 ×*g* for 15 min at 4°C and resuspended in 5 ml of lysis buffer (50 mM Tris-Cl, pH 8.0, 200 mM NaCl). Cell disruption was performed using sonication (Sonics & Materials, Inc., USA) for 6 min with intervals 4 sec pulses on and 6 sec off. To eliminate insoluble materials, the lysate was centrifuged at 21,000 ×*g* for 1 h at 4°C, followed by filtration through a 0.22 μm membrane. The clarified supernatant was then applied to 500 μl of Ni-NTA agarose resin (Qiagen, Germany) for affinity purification of the target proteins. The purified LysG77YL and LysG77YL_EAD proteins were subsequently stored in lysis buffer at -80°C until further use.

### Lytic Activity Assay

The lytic activity of engineered LysG77YL and LysG77YL_EAD against bacterial cells were assessed through turbidity reduction assays, following previously described methods [20]. The decrease in OD_600_ was measured every 5 min over a 60 min period at room temperature using a multimode microplate reader (SpectraMax i3x, Molecular Devices, USA). To determine the lytic spectrum, 2 μM of LysG77YL or LysG77YL_EAD were added to bacterial cells in reaction buffer (20 mM Tris-Cl, pH 8.0). The lytic range of LysG77YL and LysG77YL_EAD was represented by the relative lytic activity (%) using Eq. (1).

Relative lytic activity (%) = [(OD_600_ of control - OD_600_ of the endolysin treatment) / (the initial OD_600_ of the control)] × 100 (%) (1)

Additionally, to evaluate the minimal effective concentration, *B. licheniformis* JCM 2505 cells were treated with varying concentrations of endolysin (1, 2, and 4 μM) at room temperature for 60 min.

### Endolysin Stability Test

Endolysin stability under different conditions was evaluated using previously reported methods [20]. To assess the effect of pH on the lytic activity of LysG77YL_EAD, 2 μM of the enzyme was added to *B. licheniformis* KACC 12673 cells suspended in Britton–Robinson universal buffer (40 mM each of boric acid, phosphoric acid, and acetic acid; pH adjusted with NaOH). Relative lytic activity at various pH levels was determined using Eq (2).

Relative lytic activity = (Each condition (initial OD_600_ – 30 min OD_600_)) / (Maximal activity condition (initial OD_600_ – 30 min OD_600_)) (2)

The effect of NaCl was evaluated by adding varying concentrations of NaCl to the reaction buffer, and relative activity was calculated using Eq. (2). Thermal stability was examined by incubating the enzyme at different temperatures for 10 min, following by lysis assays. The relative activity was calculated using Eq. (2).

### Food Application in Milk

The antibacterial efficacy of LysG77YL_EAD in milk was assessed according to previously described methods with slight modifications [28]. To assess the applicability of LysG77YL_EAD in milk, B licheniformis KACC 12673 was inoculated into milk at final concentrations of 10^8^ and 10^4^ CFU/mL. Subsequently, 4 μM of LysG77YL_EAD was added. Samples were incubated at 4°C for 2.5 h, and viable cells were enumerated at 30 min intervals by plating 10-fold serial dilutions on Tryptic Soy Agar (BD Difco) and incubating at 37°C for 18–24 h.

## Results and Discussion

### Isolation and Characterization of *B. licheniformis*-Infection Bacteriophage G77YL

**Fig. 1:**
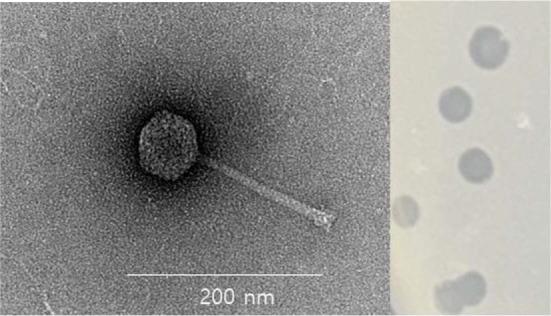
Transmission electron microscopy image and plaque morphology of phage G77YL. The scale bar represents 200 nm.

The *B. licheniformis* bacteriophage G77YL was isolated from the Gyeongchun Line Forest Trail using *B. licheniformis* KACC 18777 as a host strain. The phage G77YL formed clear plaques on double-layer agar plate with 0.7% agar in the overlay (Fig. 1) [29]. TEM analysis revealed that the GY77YL phage belongs to the class *Caudoviricetes*, featuring an isometric head with a diameter of 65 nm and a non-contractile tail approximately 200 nm in length (Fig. 1). To determine the host range, plaque assays were conducted using 26 bacterial strains, including both Gram-positive and Gram-negative bacteria. As shown in Table 1, phage G77YL exhibited host specificity toward *B. licheniformis*, forming clear plaques on the bacterial lawns of six tested strains. In contrast, no plaque formation was observed for *B. licheniformis* KACC 12673 and JCM 2505. Additionally, other Gram-positive or Gram-negative bacterial strains were resistant to G77YL phage. The host range of the newly isolated bacteriophage G77YL infecting *B. licheniformis* was consistent with a previous study [16]. The high host specificity of bacteriophages allows for the targeted elimination of pathogenic bacteria without disrupting beneficial microbiota, making them attractive candidates for applications in food safety and antimicrobial therapy [30]. In support of this, G77YL exhibited high host specificity and may serve as a potential candidate for the development of biocontrol agents targeting *B. licheniformis*.

### Genome Analysis of Bacteriophage G77YL

**Fig. 2:**
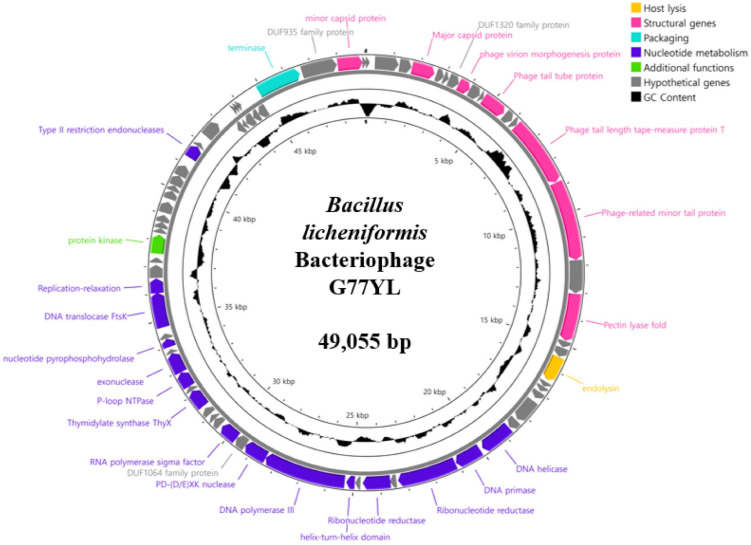
Circular representation of the *B. licheniformis* bacteriophage G77YL genome. The outer circle indicates the predicted open reading frames (ORFs) by each strand. The functional categories are indicated by specific colors according to the legend. The inner circle with the black line indicates the GC content. The scale units are base pairs.

Whole genome sequence analysis of *B. licheniformis* phage G77YL revealed that its genome consists of 49,055 base pairs of double-stranded DNA with a GC content 43.1%, containing 71 predicted ORFs and lacking tRNA genes. The predicted ORFs encode host lysis (endolysin), structural genes (capsid protein, phage tail protein, and phage virion protein, and pectin lyase), packaging (terminase), nucleotide metabolism (DNA helicase, DNA primase, ribonucleotide reductase, DNA polymeras III, thymidylate synthase, P-loop NTPase, exonuclease, nucleotide pyrophosphohydrolase, DNA translocase and Type II restriction endonucleases) and additional functions (protein kinase) (Fig. 2). Based on these functions, G77YL phage possesses its own replication and host lysis systems, enabling phage genome replication and cell burst. No lysogeny-related genes such as genome attachment site(*attP*) and integrase were detected in the genome of G77YL phage, indicating that G77YL phage is virulent phage [31]. Furthermore, the absence of virulence factors or toxin genes suggests that this phage may be a suitable candidate for use as a biocontrol agent. Comparative genomic analysis using BLASTN revealed that the whole genome sequence of the G77YL phage exhibited high similarity to *B. licheniformis* phages, including phage Jayan (PQ299153.1), PK-1(PP552876.1) and PS1 (PQ758588.1), showing 95.52% identity with 97% query coverage, 97.67% identity with 97% query coverage, and 97.97% identity with 99% query coverage, respectively. To investigate the evolutionary relationship of G77YL, a phylogenetic tree was constructed using complete genome sequences of 17 *Bacillus* phages sharing over 70% sequence identity (Fig. S1). The analysis demonstrated that G77YL clustered with *B. licheniformis* phage PS1 and showed close relatedness to *B. subtilis* phages.

### Identification and Overexpression of G77YL Phage Endolysin

**Fig. 3:**
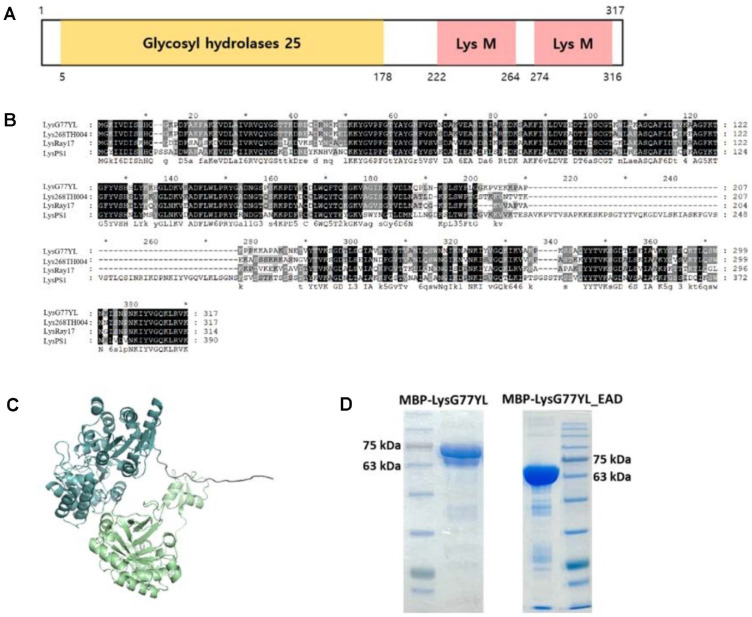
Molecular structure of LysG77YL. (**A**) Schematic representation of LysG77YL. The protein consists of an N-terminal catalytic domain belonging to the glycosyl hydrolase family 25 (GH25; 5–178 amino acids) and C-terminal region contains two LysM domains (222–264 and 274–316 amino acids). (**B**) Amino acid sequence alignment of LysG77YL related endolysins (Lys268TH004, LysRay17, and LysPS1). (**C**) Predicted three-dimensional model of recombinant LysG77YL. N-terminal maltose-binding protein (MBP) fusion shown in cyan and LysG77YL in light green. (**D**) Expression and purification of MBP-LysG77YL and MBP-LysG77YL_EAD proteins.

The 954 bp endolysin gene, classified as glycosyl hydrolases family 25 gene, was identified in the whole genome sequence of phage G77YL and designated as LysG77YL. The domain architecture of LysG77YL was predicted using InterProScan analysis (Fig. 3A). The protein contains a glycosyl hydrolase family 25 (GH25) domain in its N-terminal region (5-178 amino acids), followed by an internal linker region (179-221 amino acids), and two consecutive LysM domains located in the C-terminal region (222-264 and 274-316 amino acids). BLASTP analysis revealed that LysG77YL exhibits high protein sequence similarity, showing 80.44% identity with Lys268TH004 (QQO40378.1), 71.16% identity with LysRay17 (YP_010644211.1) and 99.37% identity with LysPS1 (XPP56484.1) (Fig. 3B). The protein structure prediction analysis using AlphaFold2 revealed that LysG77YL displays well-defined secondary structural elements including α-helices and β-sheets, which are organized into two distinct domains (Fig. 3C). The recombinant LysG77YL protein with an N-terminal His tag (~35 kDa) was first expressed in *E. coli*, but the yield was low due to poor solubility (Fig. S2). To enhance solubility, the recombinant endolysin was fused to maltose binding protein (MBP) and subsequently purified by Ni-NTA affinity chromatography. The purified MBP-LysG77YL were observed as single bands at approximately 70 kDa on SDS-PAGE, slightly lower than predicted molecular weights of MBP-LysG77YL (79.0 kDa) (Fig. 3D). Based on previous studies that deletion of the C-terminal CBD enhances lytic activity and the enzyme activity can be mediated solely by the EAD [32], a recombinant MBP-LysG77YL_EAD protein lacking the CBD was also constructed. SDS–PAGE analysis revealed a distinct band at approximately 68 kDa corresponding to MBP-LysG77YL_EAD, confirming successful overexpression and purification of the recombinant protein (Fig. 3D).

### Antimicrobial Activity of LysG77YL and LysG77YL EAD

**Fig. 4:**
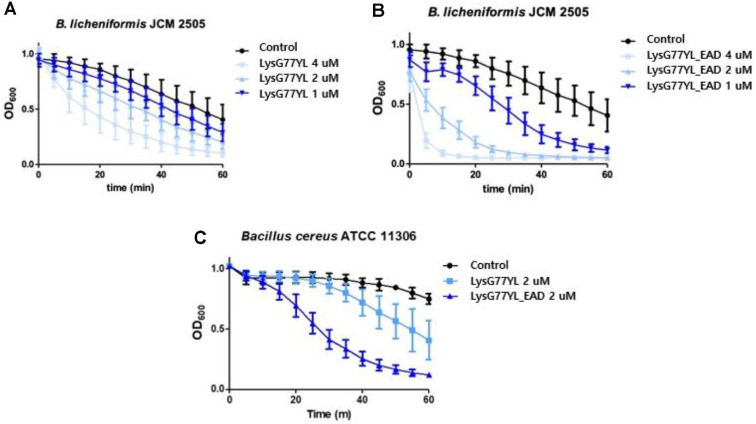
Lysis of *B. licheniformis* JCM 2505 treated with three different concentrations of LysG77YL (A) and LysG77YL_EAD (B), and lysis of *B. cereus* ATCC 11306 treated with 2 μM of each protein (C). The graph represents the means and standard deviations (SDs) from triplicate assays.

To evaluate antimicrobial spectrum of LysG77YL and LysG77YL_EAD, lytic activity assay was performed with 8 *B. licheniformis* strains and other Gram-positive and Gram-negative bacteria. LysG77YL_EAD exhibited lytic activity against all 18 tested *Bacillus* strains, including 8 *B. licheniformis*, whereas LysG77YL lysed only 12 strains, including the same 8 *B. licheniformis*, with relatively lower activity (Table 1). These results suggest that endolysins, particularly those containing an EAD, possess broader and stronger antibacterial activity than phages, as they directly target and degrade the peptidoglycan layer of the bacterial cell wall [33]. LysG77YL_EAD exhibited strong lytic activity and demonstrated high specificity toward *Bacillus* species, including the foodborne pathogen *Bacillus cereus*, while showing no lytic activity against other Gram-positive bacteria such as *Listeria monocytogenes*, *C. perfringens*, *Lactobacillus plantarum*, and *Bifidobacterium adolescentis* (Table 1). Both LysG77YL and LysG77YL_EAD exhibited concentration-dependent lytic activity against *B. licheniformis* JCM 2505 (Fig. 4A and 4B). Notably, incubation with 4 μM LysG77YL_EAD reduced the turbidity of the bacterial suspension to the baseline detection level within 5 min (Fig. 4B). Furthermore, when the lytic activities of LysG77YL and LysG77YL_EAD were compared at the same concentration against *B. cereus* ATCC 11306, LysG77YL_EAD demonstrated a superior lytic effect relative to LysG77YL (Fig. 4C). These findings indicate that enhanced efficacy may be due to the absence of the CBD, which can limit enzyme diffusion by binding it to lysed bacterial cells. In the absence of CBD, the EAD is likely to diffuse more freely, thereby increasing its access to target sites and enhancing lytic activity against Gram-positive bacteria [34]. As LysG77YL_EAD induced rapid bacterial lysis and exhibited a significantly broader lytic spectrum than LysG77YL, it was further characterized as a potential biocontrol agent against *B. licheniformis*.

### Application of LysG77YL_EAD in Commercial Milk

**Fig. 5:**
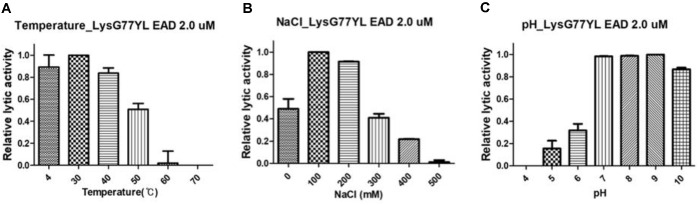
Stability of LysG77YL_EAD against *B. licheniformis* KACC 12673 (OD_600_ = 0.6) under various temperature, NaCl concentration and pH condition. Each column represents the mean of triplicate experiments, and error bars indicate standard deviations (SDs).

Considering the diverse temperatures, NaCl concentrations, and pH levels present in food-related environments, evaluating the stability of phage-derived agents under such conditions is essential for their practical application. To determine the stability of LysG77YL_EAD, the assay was examined under broad ranges of temperature (4°C to 70°C), NaCl concentrations (0 mM to 500 mM), and pH conditions (4 to 10) against *B. licheniformis* KACC 12673 (Fig. 5). The thermostability of LysG77YL_EAD were retained, maintaining 80% of its lytic activity after 10 min incubation at 4°C to 40°C. However, its lytic activity decreased when incubated at 50°C or higher. The relative lytic activity of LysG77YL_EAD were maximal in the presence of 100 mM to 200 mM NaCl, after which it gradually decreased at concentrations higher than 300 mM. Additionally, LysG77YL_EAD exhibited the highest lytic activity in the neutral to alkaline pH range (pH 7.0–9.0). Accordingly, LysG77yL_EAD demonstrated modest stability across a broad range of these factors, highlighting its potential suitability for use in complex food matrices.

**Fig. 6:**
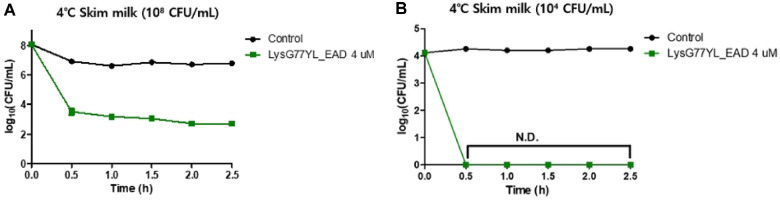
Food application of LysG77YL_EAD in milk containing *B. licheniformis* KACC 12673. (**A**) *Bacillus licheniformis*-contaminated milk sample (10^8^ CFU/ml) and (**B**) *B. licheniformis*-contaminated milk sample (10^4^ CFU/ml) treated with LysG77YL_EAD (4 μM). The values are the means and standard deviations (SDs) from triplicate assays.

The efficacy of LysG77YL_EAD in controlling *B. licheniformis* contamination in food was evaluated. Since *B. licheniformis* has been reported to frequently contaminate dairy products [3, 4], sterilized milk was chosen to evaluate the *B. licheniformis*-specific inhibitory activity of LysG77YL_EAD in food. Purified LysG77YL_EAD (4 μM) was added to the milk samples containing two different concentrations (10^8^ CFU/mL and 10^4^ CFU/mL) of *B. licheniformis* KACC 12673, and the mixture was incubated 4°C. During 30 min of incubation, treatment with LysG77YL_EAD in *B. licheniformis*-contaminated milk resulted in a 4 log reduction in the viable cell count of *B. licheniformis* compared to the untreated control.(Fig. 6). Although phage-based studies have been reported for the biological control of *B. licheniformis* in milk [16], no studies to date have reported the application of endolysin for the control of *B. licheniformis* in dairy products. In this regard, this study is the first demonstrate the feasibility of endolysin application against *B. licheniformis* within dairy matrices. Moreover, the inability of bacteriophage G77YL to infect *B. licheniformis* KACC12673 and JCM 2505 likely reflects host receptor specificity, whereas the endolysin G77YL_EAD directly hydrolyze peptidoglycan in a receptor-independent manner (Table 1). This is an advantage of endolysins in overcoming host range limitations inherent to bacteriophages. These findings suggest that LysG77YL_EAD has the potential to effectively control *B. licheniformis* and other diary-associated spoilage bacteria, thereby enhancing the reliability and efficacy of natural antimicrobial treatments in dairy products.

## Supplemental Materials

Supplementary data for this paper are available on-line only at http://jmb.or.kr.



## Figures and Tables

**Table 1:** Host range of phage G77YL and lytic activity of LysG77YL and its enzymatic active domain LysG77YL_EAD.

Species	Strain no.	G77YL^a^	Relative lytic activity^b^	
			LysG77YL	LysG77YL_EAD
*Bacillus licheniformis*	KACC 12673	-	+	++
	KACC 15827	+	+	++
	KACC 15832	+	+	+
	KACC 15835	+	+	+
	KACC 15896	+	++	++
	KACC 18777	+	+	+
	JCM 2505	-	+	++
	HNIBR-BC 151	+	+	+
Other Gram-positive				
*Bacillus cereus*	ATCC 10987	-	+	++
	ATCC 11306	-	-	++
	ATCC 21772	-	-	+
	ATCC 27348	-	-	++
	NCCP 10821	-	-	+
	NCCP 11309	-	-	+
*Bacillus amyloliquefaciens*	KACC 10116	-	-	+
*Bacillus subtilis*	ATCC 23857	-	+	+
*Bacillus pumilus*	JCM 2508	-	+	+
*Bacillus velezensis*	HINBR-BC323	-	+	+
*Listeria monocytogenes*	ATCC 15313	-	-	-
*Clostridium perfringens*	FD1	-	-	-
	ATCC 13124	-	-	-
	NCCP 15911	-	-	-
*Lactobacillus plantarum*	ATCC 8014	-	-	-
*Bifidobacterium adolescentis*	ATCC 15703	-	-	-
Other Gram-negative				
*Escherichia coli* O157:H7	ATCC 35150	-	-	-
*Salmonella* Typhimurium	UK1	-	-	-

^a^+, activity of dotting assay; -, no activity.

^b^-, 0-10% activity; +, 11-40% activity; ++, 41-70% activity; +++, 71-100% activity
