# Supplementary material for: Characterization of Endolysin LysG77YL from Bacillus licheniformis-Infecting Bacteriophage G77YL and Application as an Antimicrobial Agent
Source: J Microbiol Biotechnol. 2026 Mar 25;36:e2601064. doi: 10.4014/jmb.2601.01064 (PMC13036505; doi:10.4014/jmb.2601.01064)
Supplement: Supplementary file 1 [file jmb-36-e2601064-supple.pdf]

J. Microbiol. Biotechnol. 2026. 36: e2601064

https://doi.org/10.4014/jmb.2601.01064

Supplementary Table and Figures

Table S1. Plasmids and primers used in this study.

Plasmids or primers	Description or sequences (5′ to 3′)
**Plasmids**	
pET28a	pET28a Kan^r
pET28a::MBP::LysG77YL	pET28a Kan^r; MBP; LysG77YL (BamHI / HindIII)
pET28a::MBP::LysG77YL_EAD	pET28a Kan^r; MBP; LysG77YL_EAD (BamHI / HindIII)
**Primers**	
fBamH_ LysG77Y _N	gcg GGATCC ATGGGTAAAATCGTTGACATTTCACACC
rHind_ LysG77YL_N	gcg AAGCTT TTACTTGACTCGTAGCTTCTGACCAACGTA
F_BamH_N_G77YL_EAD	gcg GGATCC ATGGGTAAAATCGTTGACATTTCACAC
R_Hind_N_G77YL_EAD	gcg AAGCTT TTATTTCTTTGGCGGCTTTGGAG

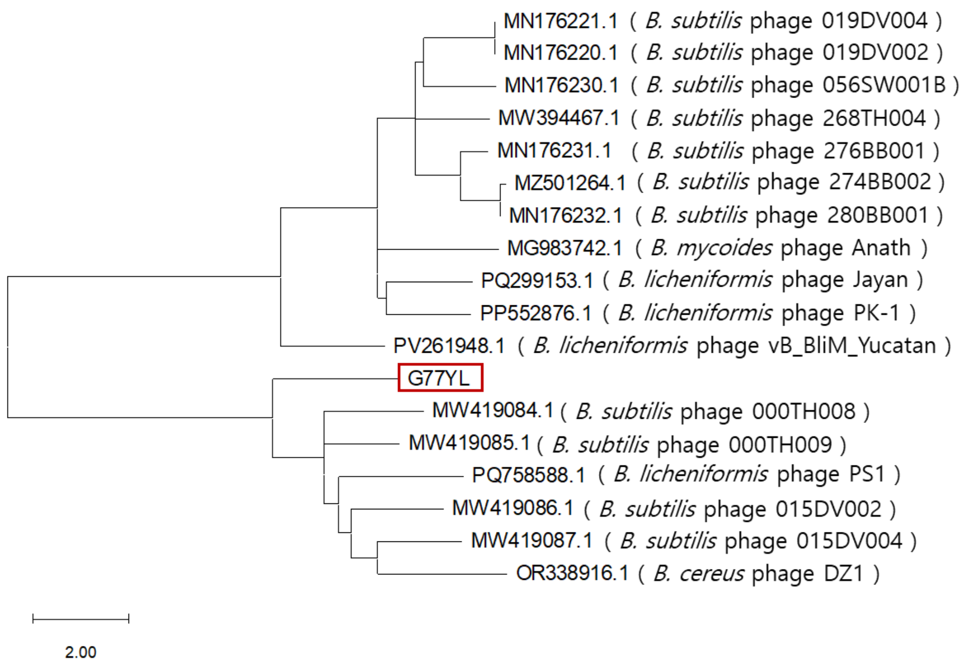

Fig. S1. A phylogenetic tree of phage G77YL constructed using complete genome sequences of related *Bacillus* phages.

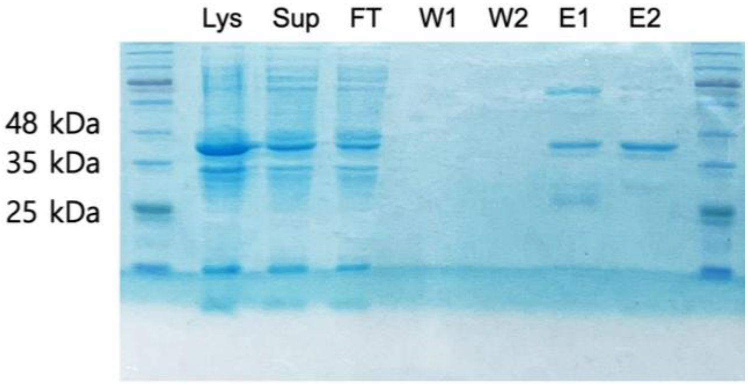

Fig. S2. Expression and purification of LysG77YL by SDS-PAGE. Lys, whole-lysate; Sup, supernatant; FT, flow-through; W1 and W2, wash buffer; E1 and E2, elution samples. The LysG77YL protein was observed at approximately 35 kDa.
